# Medicine Drug Name Detection Based Object Recognition Using Augmented Reality

**DOI:** 10.3389/fpubh.2022.881701

**Published:** 2022-04-29

**Authors:** Ch. Rupa, Gautam Srivastava, Bharath Ganji, Sai Praveen Tatiparthi, Karthik Maddala, Srinivas Koppu, Jerry Chun-Wei Lin

**Affiliations:** ^1^Velagapudi Ramakrishna Siddhartha Engineering College, Vijayawada, India; ^2^Department of Mathematics and Computer Science, Brandon University, Brandon, MB, Canada; ^3^Research Centre for Interneural Computing, China Medical University, Taichung, Taiwan; ^4^School of Information Technology, Vellore Institute of Technology, Vellore, India; ^5^Department of Computer Science, Electrical Engineering and Mathematical Sciences, Western Norway University of Applied Sciences, Bergen, Norway

**Keywords:** augmented reality, surface view, Drug name recognition (DNR), rendering, Sceneviewer, ArCore, 3D model

## Abstract

Augmented Reality (AR) is an innovation that empowers us in coordinating computerized data into the client's real-world space. It offers an advanced and progressive methodology for medicines, providing medication training. AR aids in surgery planning, and patient therapy discloses complex medical circumstances to patients and their family members. With accelerated upgrades in innovation, the ever-increasing number of medical records get accessible, which contain a lot of sensitive medical data, similar to medical substances and relations between them. To exploit the clinical texts in these data, it is important to separate significant data from these texts. Drugs, along with some kind of the fundamental clinical components, additionally should be perceived. Drug name recognition (DNR) tries to recognize drugs specified in unstructured clinical texts and order them into predefined classifications, which is utilized to deliver a connected 3D model inside the present reality client space. This work shows the utilization of AR to give an active and visual representation of data about medicines and their applications. The proposed method is a mobile application that uses a native camera and optical character recognition algorithm (OCR) to extract the text on the medicines. The extracted text is over and above processed using natural language processing (NLP) tools which are then used to identify the generic name and category of the drug using the dedicated DNR database. The database used for the system is scraped using various resources of medical studies and is named a medi-drug database from a development standpoint. 3D model prepared particularly for the drug is then presented in AR using ArCore. The results obtained are encouraging. The proposed method can detect the text with an average time of 0.005 s and can produce the visual representation of the output with an average time of 1.5 s.

## Introduction

Augmented Reality (AR) blends both real-world and virtual-world, which are artificially generated. It assists in seeing 3D virtual articles superimposed upon the present reality by using an advanced data view (1). AR is utilized in many territories, including healthcare, instruction, assembling, and diversion. Especially in the clinical area, with the progression in optics, PC frameworks, and careful instruments, the utilizations of AR in medication are energetically investigated. The extent of its progression in the clinical field depends on the clinical fortes, specialized effects, development of ventures, and distributions on the subject of AR in healthcare. Before the advent of mobile phones, there were limited advancements in AR applications (2). With the innovation of handheld gadgets like cell phones and tablets, AR progressed to cater greater audience. In 2013, tech company Google introduced Google Glass, an HMD that gives a voice interface and empowers clients to call, send messages, and search the web. In 2015, tech giant Microsoft introduced the HoloLens; a gadget that permits one to interface with holographic 3D virtual items utilizing voice, look, and motions (3, 4).

Nowadays, AR has turned into a vastly examined topic and an excellent field for new sorts of applications in the clinical area. In addition, this technology has opened up opportunities in the field of healthcare. Innovations in AR improve the abilities of doctors and surgeons to treat, diagnose (5), and perform surgeries on patients even more precisely because of the real-time data and information of the patients they could have access to Gadekallu et al. (6), Rizwan et al. (7), and Reddy et al. (8). It can also be an effective learning way for the students and trainees as the visuals of dealing with various health issues and scenarios, created through AR can help them understand quite easily (9, 10). AR brings ground-breaking benefits to the healthcare industry, and we are currently witnessing the beginning of AR in medicine.

Augmented reality is revolutionizing the way doctors perform their operations. Examination of an affected body part has become easy with 3D X-Rays. It has also become way easier to treat the tumors as they can be seen in 3D, with patients not needing to undergo any radiation-related process for tumor detection (11). Devices facilitated with AR and their applications will soon be in utilization for every patient treatment (12, 13). AR apps would be considered for pain management, and they would be more accessible. AR applications could be useful in such areas as rehabilitation of various kinds, neuropathic pain, and also mental health treatment. AR is evolving faster, better, and even meeting the day-to-day routine of the healthcare industry (14). AR can change the clinical business and in the upcoming decade, we can see many progressed systems coming into the general public that could well revolutionize the medical industry. The proposed work centers around a better approach to access data about the employment of a specific medication. With the availability of the web, data about anything is accessible and the data about restorative medications is not a special case. Our work proposes a straightforward and visual approach for portraying the use of a therapeutic medication utilizing AR innovation. The proposed work takes the unstructured text of the clinical names and conveys the data regarding that specific medication as a 3D model in AR. In the entire system, the designing of any module is independent of other modules and they collate together while producing the desired output. This helps the clients to effectively and more significantly comprehend the utilization and use of restorative medications. It helps the clients to look physically on the web and go through various site pages to comprehend the medication as the proposed work requires the client to simply bring up the therapeutic message to the camera of the cell phone. The rest of the paper is organized as follows. Section 2 discusses associated work along with a summary by considering some properties. The proposed methodology to detect drug-using AR was discussed in Section 3. Moreover, it consists of details about various phases of the proposed system functionality. Section 4 discusses the results and analysis along with a comparative study.

## Related Work

Oufqir et al. (15) discussed ARKit and ArCore, the two open-source libraries that show virtually created models in reality. The additional information provided about the scene, object, and the standard development kit (SDK) are generalized for all categories of devices like smartphones, tablets, etc. However, the discussion hasn't mentioned how the scene is detected and how the information is processed.

Muñoz-Saavedra et al. (2) proposed work-focused healthcare, which is the most notorious field of application of AR and VR. The authors noticed that the most significant subtopic is a medical methodology, followed eagerly by mind disquisition and rehabilitation. In this composition, an all-around examination of various fields which is offered by computer-produced augmented commands is exploited. This study helps in creating the most advised models for presenting to users.

Yung and Khoo-Lattimore (3) discussed the role of AR in tourism. Regardless of the developing interest and conversations on AR and VR in the tourism business, it is not deliberately known whether the data has been answered from scholarly documents on VR and AR. By driving an intelligent composing review on VR/ AR exploration in the trip assiduity area, this work attempts to reply to five essential exploration questions. Which of the movement business regions and settings have VR and AR exploration addressed? Which sorts of VR and AR have collected the important study in the movement of business exploration? What ways/ propositions are being employed to probe VR and AR in the movement business? Also, what are the research gaps in VR and AR tourism research? From a combination study of 46 manuscripts, marketing and tourism education, awareness on the technology and usability was increased in the most recent time period.

Aggarwal and Singhal (9) talked about AR, which is a mix of objects and a computer-created or virtual world. It is accomplished by increasing computer-processed and created pictures in reality. It is of four varieties to be specific, marker-based, projection-based, markerless, and super-imposition-based AR. It has numerous applications in reality. AR is utilized in different fields like medical, training, assembling, mechanical technology, and diversion. Increased reality goes under the field of mixed reality.

Huang et al. (16) developed a sign-perusing assistant which recognizes digital word text and converts it to highly computed AR lettering. This framework proposes an assortment of navigational instruments accessible to help with this visual data task. This expected methodology provides upgraded visual data about the area and the substance of existing signs in the climate. This framework fosters a model programming application that facilitates sudden spikes in demand for head-mounted expanded reality gadgets which can help clients to help with sign perusing. This proposed method helps in detecting real-time information but it is equipped with a lot of hardware and the methodology need to be simplified for populating information to the user.

Guerrero et al. (17) proposed a system on intelligent AR for medical assisting management. This system focused on the advancement of clever assistive frameworks that offer individual help to people. By utilizing the formal argumentation hypothesis, this framework has fostered a projection-based increased reality in the UI. This proposed system helps to detect medicine pills and project the information regarding the pills through AR.

Namysl et al. (18) proposed an optical character recognition (OCR) system combined with deep learning which provides a solution for detecting text in unconstrained environments. For building the system, they have to render synthetic training data using large text corpora and over 2,000 fonts. This proposed method was limited to the detection of text and had not presented any methodology for linking it to AR.

Qiao et al. (19) proposed portable mobile expanded Reality (Mobile AR) that has begun getting expanded consideration from both industry and the scholarly world. Application-based Portable AR and Hardware-based Mobile AR are the two winning stages for Mobile AR applications. In any case, equipment-based Mobile AR executions are exorbitant, and it needs adaptability. Simultaneously, the App-based one requires extra downloading and establishment ahead of time and is awkward for cross-stage organizations. Besides, the experts guess that Web AR will convey inventive innovation to supplement our approaches to communicating with the physical (and digital) world around us and at a comparable time.

Lin et al. (20) discussed a text detection and recognition approach in a scene using computer vision. The work depicts a comparison between different text recognition algorithms that were made and the results are illustrated diagrammatically. The work does not provide any novelty for either a new application or a new method of invention.

Flores-Flores et al. (21) proposed an engineering approach for the incorporation of AR in connected open medication information. The fast increment of cell phones and the steady improvement of their taking care of capacities have helped the improvement of different advancements, like AR. Then again, there are as of now more associations that make their data accessible to society, as the drug business, this helps the development of the Linked Open Drug Data (LODD). This structure Augmented Reality in Linked Open Data cloud (ARLOD) helps in cultivating an application for handheld devices that joins AR information and the LODD datasets.

Park et al. (22) proposed a profound learning module based on the projection AR system for medical help and to work on the government assistance of the old. The proposed work comprises posture assessment, face acknowledgment, and item recognition modules. By utilizing these modules, a bidirectional creation increased reality framework which gives significant data by understanding the client environment. Deep learning frameworks along with projection-based AR were effectively implemented for detecting text and projecting an augmented model.

Knopp et al. (23) addressed the drawbacks in the use of HoloLens, in scenarios where it is hard to track objects of similar shape. This approach empowers the choice to involve the HoloLens in a truly adaptable climate along with current innovations like AI. The adaptability is restricted to a specific equipment level.

Chaithanya et al. (24) proposed a Text location in regular pictures calculation which plays a fundamental part in the field of increased reality. It assists in eliminating commotion in pictures and recognizes text. In any case, it is furthermore a troublesome issue due to the inconstancy in imaging conditions, for instance, lighting, secular reflections, upheaval, dark, and closeness of squares over the substance. Extraordinary text revelation computations ought to be used as needs be enthusiastic against such changes. The proposed method takes the advantage of OpenCV module for detecting text which is not frequently used in the development, so it has limited the proposed method to static images.

Armengol-Estapé et al. (25) proposed a model that includes the adaption of a cutting edge NeuroNER is a program that performs Named-entity recognition using neural networks (NER) (i.e., NeuroNER). It depends on some of the Spanish medical texts where the profound learning space is used to recognize proteins or different parts. The framework, we base our work on, has an AR separated from profound learning parts. We proposed detecting medical text and displaying related diseases. The proposed system limited the scalability to a certain hardware level. A summary of related works is given in Table 1.

**Table 1:** Summary of related works.

**References**	**Year**	**Objective**	**Pros**	**Cons**
Flores-Flores et al. (21)	2019	Reconciliation of Augmented reality in connected open medication information.	Application well suited and designed for mobile devices	Standard Development Kit is deprecated or no longer used.
Park et al. (22)	2019	A projection-based expanded augmented System along significant literacy module for the medical help and to work on the government assistance of the older	A bidirectional production-based augmented reality system which provides important data by understanding the client conditions	An external handset is required and high computation devices are required.
Knopp et al. (23)	2019	An approach for transferring algorithms like picture handling away from the restricted equipment of a transparent Head Mounted Display (HMD) like the HoloLens to an all the more impressive, far off PC that isn't fixed on the x86 engineering	Live object tracking along with text detection.	An external handset is required and not suitable for mobile applications.
Chaithanya et al. (24)	2019	based on the profound learning space of the Spanish medical texts to distinguish ways of life as proteins or different parts.	Text detection algorithm for low-quality images and natural images.	No further projection of identified text. Not suitable for handheld devices.
Armengol-Estapé et al. (25)	2019	An approach for the detection of chemical entities using machine learning and natural language processing.	Detection of text in multiple languages majorly in Spanish.	Not suitable for all mobile devices. A proper user interface is not provided.
Proposed	2022	uses a Drug name Recognition to perceive unstructured medical texts and arrange them into predefined drug classifications using an AR camera. The related augmented model is presented in the user environment.	Live detection of text on the user environment and optical characters using mobile applications. The information and augmented model are presented to the user.	Predefined data for classification of recognized drug data.

## AR-Based Drug Detection System (AR-DDS)

The proposed work uses Drug name Recognition (DNR) to recognize unstructured medical texts and group them into predefined drug classes. When a user scans the medicine sheet using an AR camera which is programmed with an OCR algorithm to identify the drug name using the surface view, the recognized text from the surface view is then compared with the drug names in the dataset which is predefined and then categorized accordingly. If the recognized text matches the drug name in the dataset, then the application will return the output by rendering the corresponding 3D Model using Scene viewer (4). The rendering can be done using predefined and well-formed libraries given by the software during the Application programming interface invocation. This AR application is used for general and medical customer service.

### Functioning of AR-DDS

**Figure 1:**
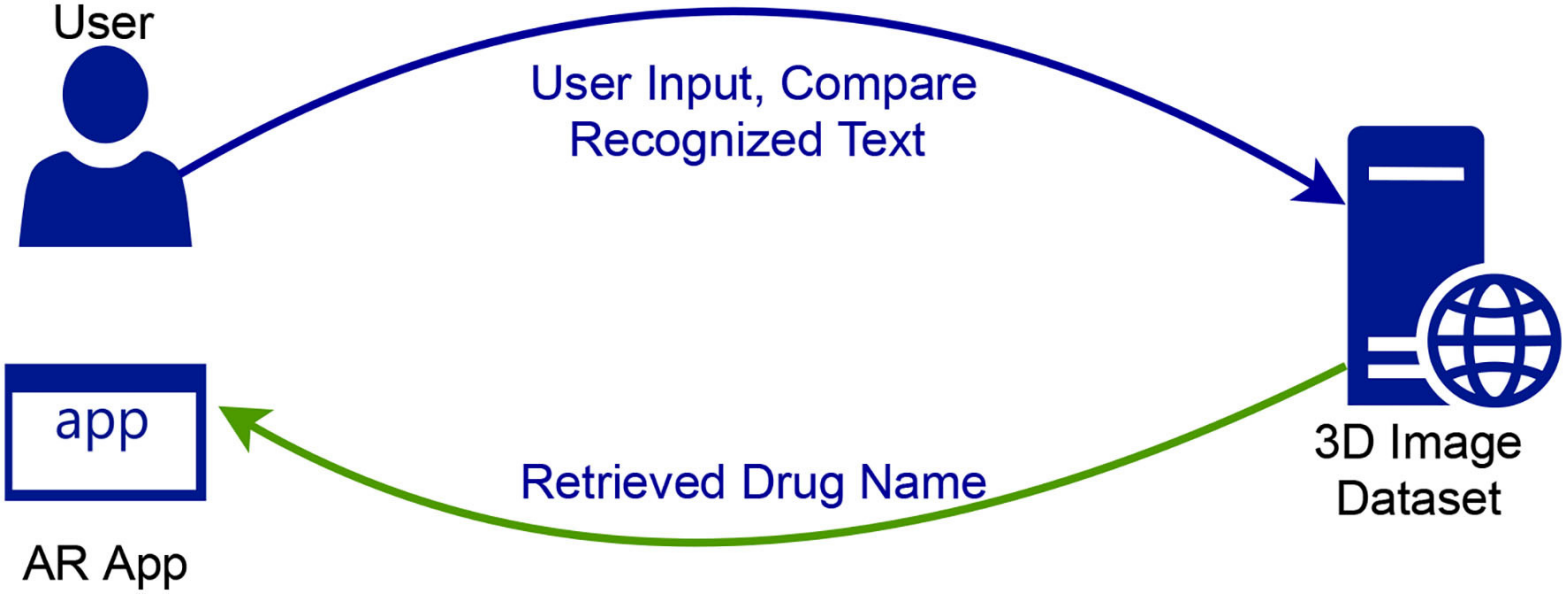
Transaction flow system mode.

Figure 1 shows the progression of exchanges among the framework parts. The proposed system uses an in-built OCR functionality of the operating system to make the text recognition. The 3D models are retrieved from the online server during runtime and rendered using Sceneviewer and along with the 3D Model, a short description of the retrieved drug name is populated.

**Figure 2:**
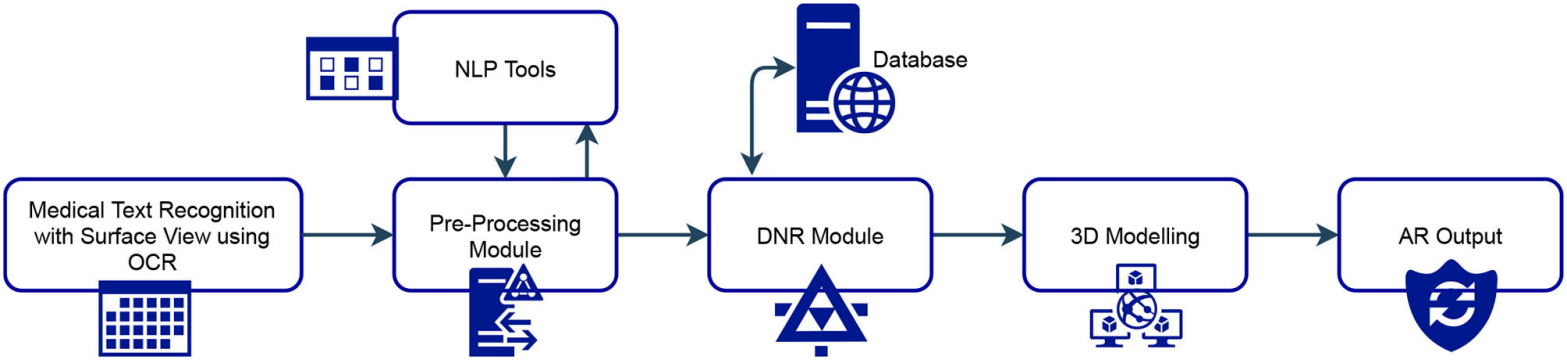
Proposed system architecture.

Figure 2 shows the proposed system architecture. Here, SurfaceView offers a drawing surface while the surface's format is often managed, SurfaceView takes care of putting the surface at the proper location on the screen. Here, SurfaceView is employed to capture images by previewing the camera onto the screen. OCR does the electronic or mechanical change of pictures (10, 26). The pictures can be composed, written by hand, or published text into machine-decoded text, regardless of print of a scene photograph, a filtered record, a report, or caption primer superimposed on a picture. An image is nothing but a group of pixels, the picture is scanned for light and dark areas, and every character is identified (27, 28).

Thus, Surface View offers to capture the medical text in the real world, and OCR runs in conjunction and converts the text observed in the SurfaceView to digital information. This process is done very efficiently in a minimal time. The text thus recognized is sent to the pre-processor module. The pre-processing module transforms the original input texts into representations and enriches the unique texts with lexical and syntactic data. Pre-handling incorporates sentence parsing, tokenization, grammatical form (POS) labeling, message lumping, and lemmatization. After text pre-processing is done, the text contains only key information that is required for drug recognition. The information could be a generic name, chemical name, product name, etc.

The pre-processed test is then fed into the DNR algorithm. DNR is a critical module that helps for the recognition of the drug. It looks to perceive drugs referenced in medical texts and characterize them into predefined classifications, which is a basic errand of medical data extraction and is a significant part of numerous medical connection extraction frameworks and applications. DNR calculation deals with a word reference-based methodology. It takes the watchwords from the pre-handled text and looks for them in the data set and gets the outcomes. 3D displaying is a PC illustration procedure for delivering a 3D advanced portrayal of any article or surface. This module helps to convert 2D images into 3D models. 3D models are then shown in AR using support libraries from Android. In the entire system, the designing of a module is independent of other modules and they collate together while producing the desired output.

### Phases of AR-DDS

Augmented Reality based drug detection (ARDS) system consists of three stages that include pre-processing, text recognition, and post-processing system. The phases of AR-DDS are combined in a single module with a separation of functionality in different functional components which communicate to each other by handing different application states and by-passing different arguments. A detailed view of individual phases is mentioned as follows:

#### Pre-processing

Among all the phases of OCR, pre-processing is the foremost and most important phase since the precision of the OCR framework relies on how well pre-handling is performed (29, 30). The fundamental target of the pre-processing stage is to make it as simple and feasible for the OCR framework to separate a person/word from the foundation. Algorithm 1 shows the steps involved. The pre-processing technique is used to improve the chances of successful recognition of images (31). Table 2 shows the pre-processing technique used and has the following operations.

**Algorithm 1:**
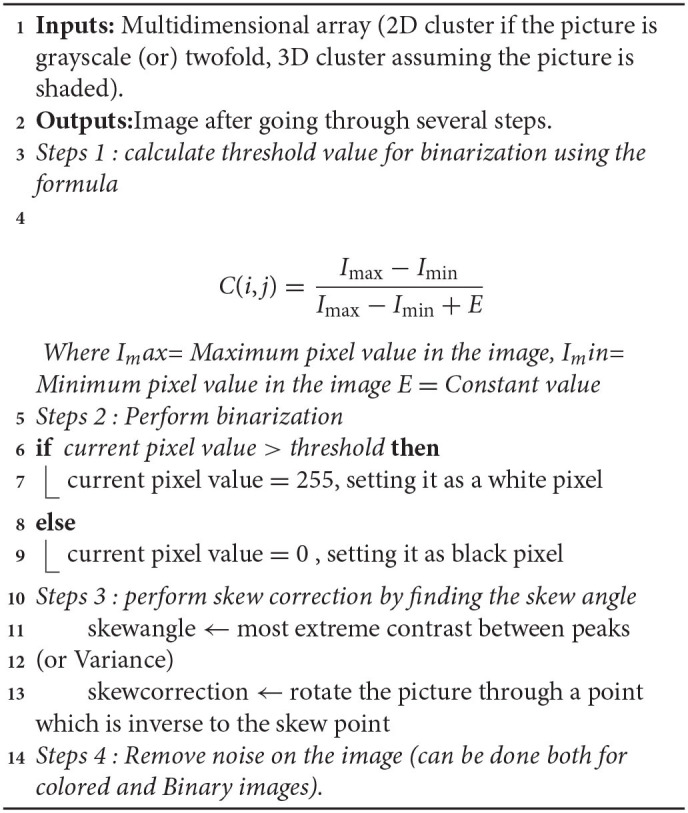
Pre-processing phase.

**Table 2:** Pre-processing technique operations.

**Operations**	**Functionality**	**Outcome**
De-skew ( )	Deskewing is an interaction by which skew is taken out by pivoting a picture by similar sum as its skew however the other way.	an evenly and in an upward direction adjusted picture where the text stumbles into the page rather than at a angle.
Despeckle ()	Despeckle just chips away at high contrast pictures, and it expects pictures that have dark text on a white foundation	more accurate OCR and barcode detection.
Binarisation ()	Binarization (thresholding) of archive pictures is the main most significant stage in pre-handling of poor-quality examined reports to save all or most extreme subcomponents like text, foundation, and picture.	Binary pictures can be gotten from dim level pictures by binarization.
Line removal ()	Finds and eliminates level and vertical lines in a 1-bit high contrast picture.	Eliminate the lines to permit more precise OCR identification.
Layout analysis ( )	Identify different rudimentary items on the picture, for example words or portions of words, separators, associated parts, shading slopes, modified text regions.	Detected objects from words.
Line and word detection ( ) ( )	Patterns for words and lines can be set.	Baseline for words
Script recognition ( )	Multilingual content may disrupt degree of words, thus used to handle the exact content.	Script after removing multilinguistic word if any.
Character isolation ( )	Different characters connected by picture antiquities ought to be broke up, unattached glyphs are broken up into a few ancient rarities-based pieces ought to be connected.	Characters after divided or characters after linking broken pieces.

#### Text Recognition

Text recognition is gainful for applications that need to recognize both individual words and series of words (1). Text recognition is involved as an independent component or on with targets. Text recognition is employed for kids' instructive games and as an apparent informative element. There are two kinds of core OCR computations, which can redeem a deposited rundown of over and upstart characters. Configuration matching includes differing an image with a put-down icon on a pixel-by-pixel supposition, additionally alluded to as “design coordinating,” “design acknowledgment,” or “picture connection.” This depends on the information glyph being accurately disconnected from the excess piece of the picture and on the put-away glyph being at a comparative text style and an identical scale. This procedure works best with typewritten text and does not function admirably when new textual styles are experienced. This is the procedure by which the primary actual photocell-based OCR is carried out, rather straightforwardly. Highlight extraction deteriorates glyphs into “highlights” like lines, shut circles, line bearing, and line crossing points. The extraction highlights lessen the dimensionality of the portrayal and make the fame cycle computationally proficient. These elements are contrasted and a theoretical vector-like the portrayal of a character, which could diminish to somewhere around at least one glyph model. Closest neighbor classifiers like the k-closest neighbor's calculation are utilized to contrast the picture included and put away glyph include and pick the nearest match. Present-day OCR programming, for example, OCR or Tesseract utilizes neural organizations that were prepared to recognize entire lines of a message rather than that represent considerable authority in single characters. Other normal configurations incorporate OCR and PAGE XML. Algorithm 2 shows the process steps.

**Algorithm 2:**
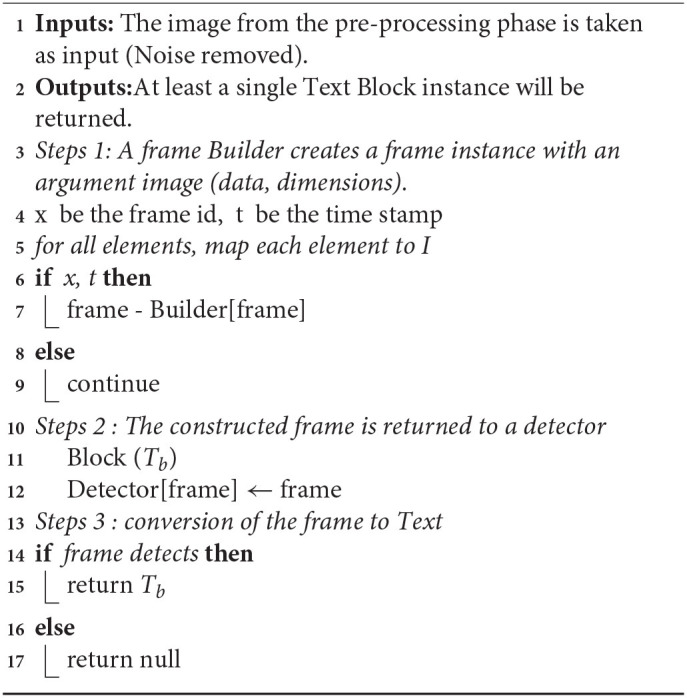
Text recognition phase.

### Postprocessing

**Figure 3:**
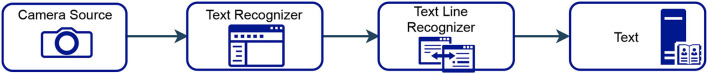
Optical character recognition (OCR) flow diagram.

Postprocessing and handling is the method that involves applying full-screen channels and impacts a camera's picture cushion before it is shown on screen. It can radically work on the visuals of the item with little arrangement time. Here, the model can utilize present handling impacts and reproduces actual camera and film properties. The post-handling step lessens the number of blunders. Post-handling rectifies each sentence in turn. The precision of the OCR can be expanded on the off chance whose result is compelled by the dictionary. This procedure could be wasteful if the record has terms not included in the dictionary, such as regular people, locations, or effects. To get further developed precision, Tesseract utilizes its term source to concussion the person division step. The resulting stream can be a plain textbook or symbols record, yet additionally, experience OCR frameworks that can keep the first design of the runner. The Levenshtein Distance computation has been utilized by OCR present handling on further enhancing developments from OCR API. The flow of actions in OCR is as shown in Figure 3. Algorithm 3 shows the process steps.

**Algorithm 3:**
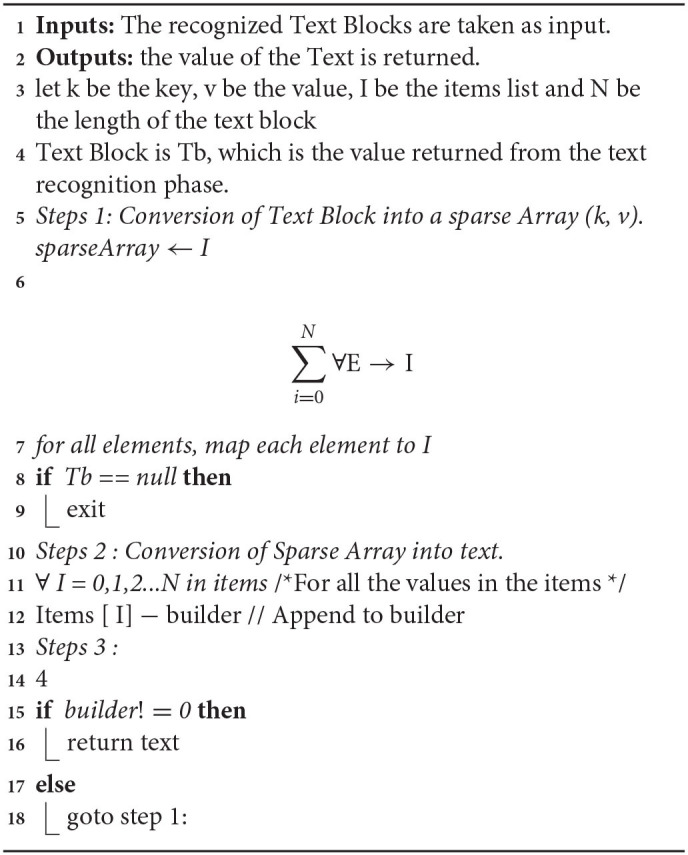
Post processing.

## Results and Analysis

The proposed Android AR-based application uses libraries and more modules and plug-ins in the plan and advancement of it. In this interaction, first, by using Android Studio, an Android project with a base API level of 24 need to be created. Then, the Android project is opened and the sceneform plug-ins need to be added. In this interaction, first, an Android project with a base API level of 24 is used. Then, the Android project is opened and the sceneform plug-ins are added, then add the following dependencies to the build. Gradle (Module: app) file in the Android project, ArCore for implementing plane area detection, sceneform, which is an animation for implementing the animations on 3D models, scene form, for assets used to build the 3D models into a renderable format, services-vision to implement OCR, to recognize text from camera source, and also add permissions to access the camera in the manifest file.

To recognize the drug name, first, there is a need to create a document where every one of the necessary bundles and modules are imported and handle the prerequisite by checking for the authorizations given by the client to access the camera by overriding the on_Request_Permissions_Result () function. Then, a camera source object is created to get the cameraview holder. The cameraview gets the input from the surface view which is sent to the Text_recognizer_file. The Text_recognizer class converts the optical characters into digital text blocks. These blocks of text are received by a detector class and converted blocks of text into individual items where the item is a set of size and value of text_lines and by using the item.getvalue() function, we can get the detected text. This text is pre-processed into a chunk of data, that is sent to the Ar.javafile. Using the AR class, there is a need to detect whether the text contains any drug name or details by using the reference of the drugdata.java class. This class contains predefined drug names and categories of the field the drug belongs to. Later to render the 3D model in AR using an object, Display Class which contains several intents to pass the 3d model to Sceneviewer. If the user demand is not satisfied, then a default Toast message is displayed on the screen with a message no model found for an identified drug name. Along with the model, a category to which the drug is related is also displayed to the user.

**Test Case 1**

**Figure 4:**
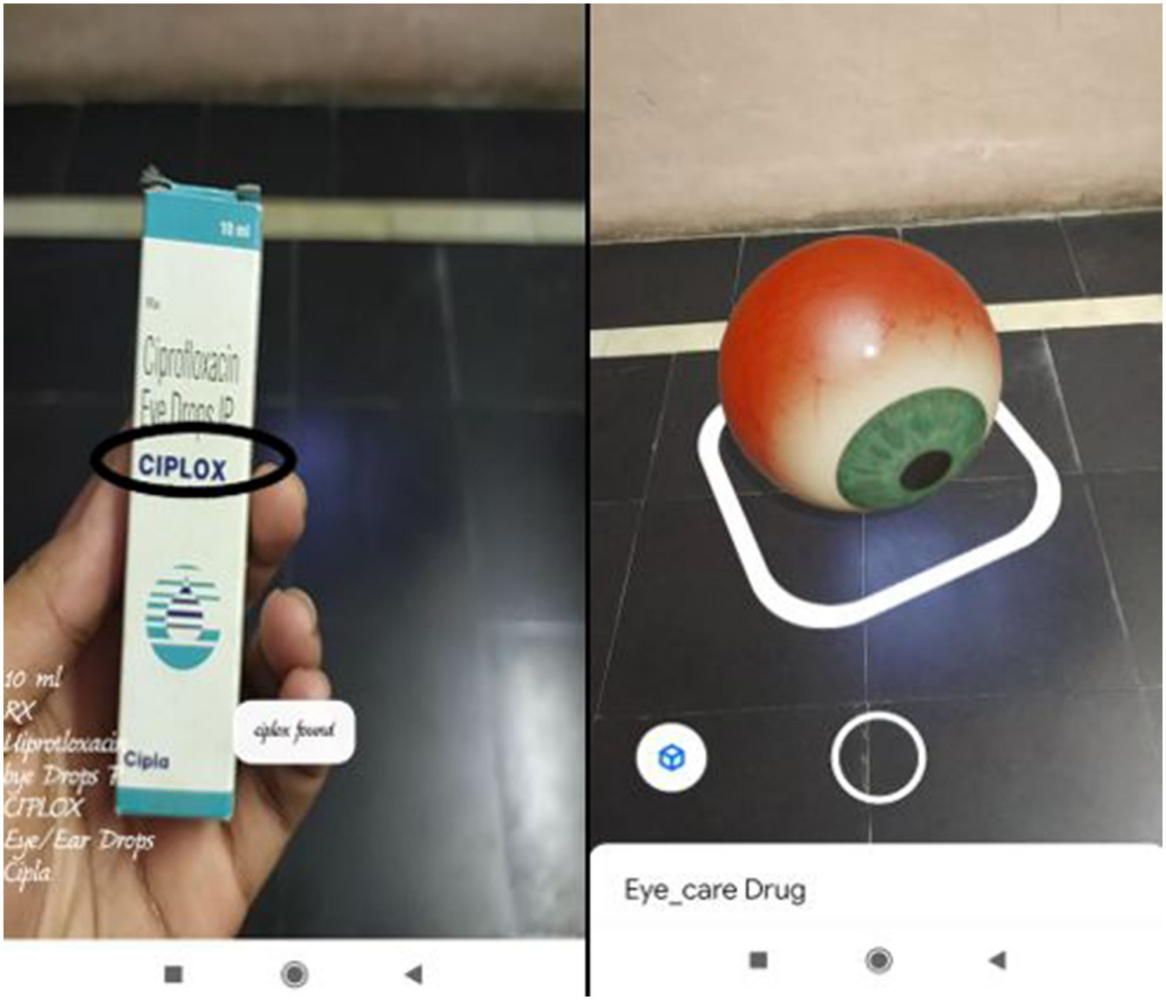
Medicine name and AR-based eye visual.

The proposed application was tested by scanning a medical sheet called ranitidine tablet which is generally used for gas trouble, and an eye drops liquid bottle called Ciplox which is generally used for common eye problems. The proposed Android application scans a particular medical sheet or drug, it detects the drug name present on the medical descriptions and gives the reactions as per the given design. The application can be utilized to identify some other drugs based on the availability of drugs in the predefined drug name as shown in Figure 4.

**Test Case 2**

**Figure 5:**
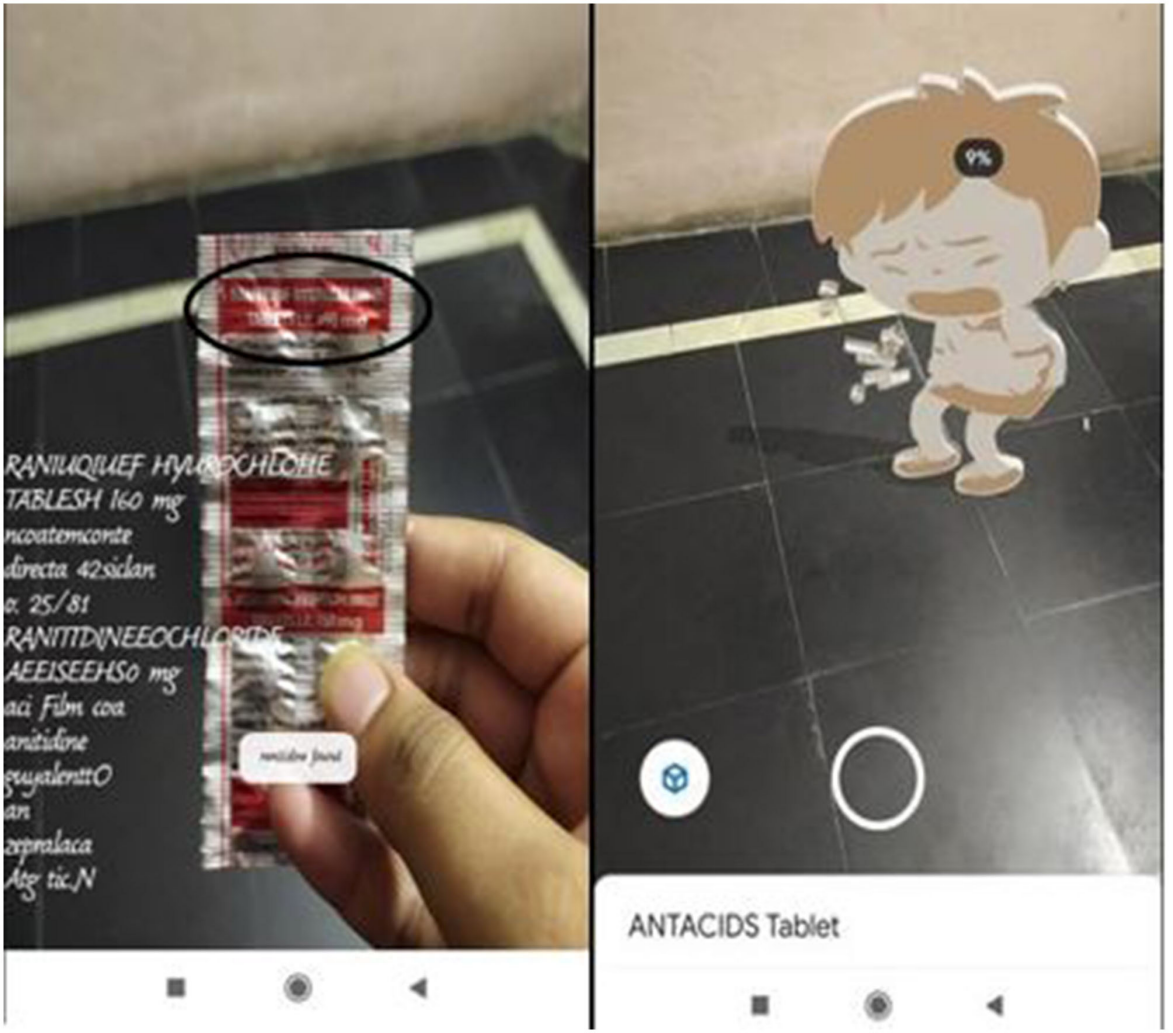
Real drug and 3D model of the drug along with name detection as ANTACIDS.

Figure 5 contains representations of both drug name detection and the presentation of the augmented 3D model. After the detection of the drug name, the application that provides a toast message of the drug name is detected and a short description or the category to which the drug belongs is also provided along with the augmented model.

### Comparative Study

The software that is used in this work is Google Cloud Vision. When we compare this particular software with some of the remaining software, like Tesseract, OCRopus, and Puma.Net, they have some drawbacks. Most of them are not online-based, i.e., installing a separate software on the PC (see Table 3). Also, OCRopus do not have a standard development kit. The Software that is used is capable of detecting almost all fonts whereas the remaining software like Tesseract, OCRopous, and Puma.net can detect a limited number of fonts. In the Proposed System, the OCR algorithm is used for better integration with the mobile platforms. By using OCR as the algorithm to detect text improves the efficiency when compared to the native method of scanning the images and later applying other strategies to extract information from the data sources. The standard development kit ArCore was used which supports most of the mobile platforms. The recommended OpenGL (Open Graphics Library) level must be greater than or equal to 3.0 and the minimum Android SDK level 24 should be met by the mobile devices. The proposed system can run on Android version 4.1 and later.

**Table 3:** Comparison among various OCR software.

**Software name**	**Online**	**SDK**	**Fonts**
Google Cloud vision	Yes	Yes	All
Tesseract	No	Yes	Any printed only
OCRopus	No	No	Normal Latin scripts
Puma.Net	No	Yes	Any printed only

### Performance Analysis

**Figure 6:**
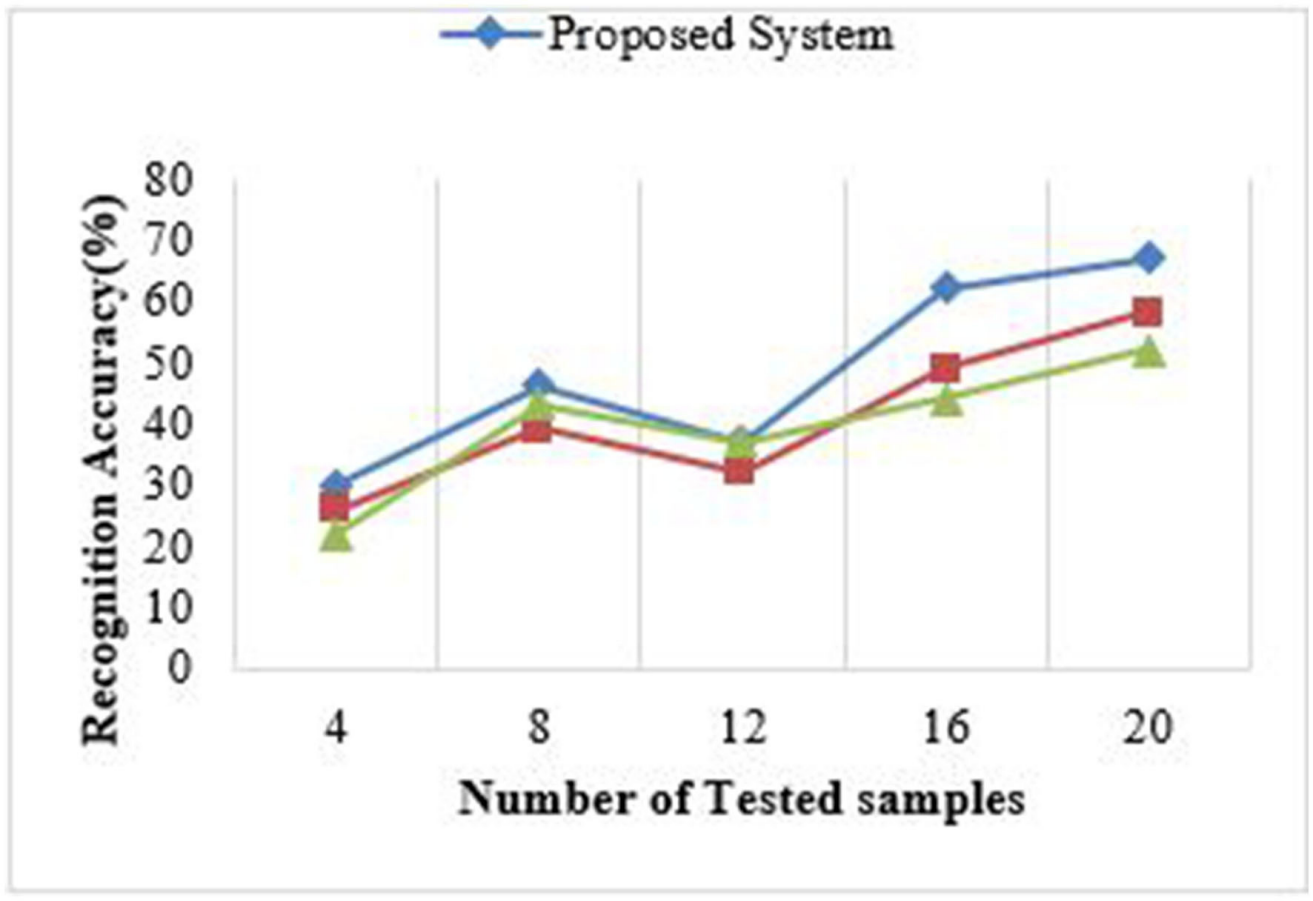
Performance analysis using functional factors.

To get the computational performance, we reformed the application execution utilizing Android Studio Logcat. This proposed framework execution is better compared to existing algorithms like Tesseract (23) and puma.net for the same sets of samples. Preprocessing, size, and resolutions of the samples, post-processing, and time are the important factors to the Android application. The text recognition accuracy is important due to the high computation power of the Android system and class files written in java, which is converted to dex files internally. The proposed system is scalable and time-efficient as shown in Figure 6.

**Figure 7:**
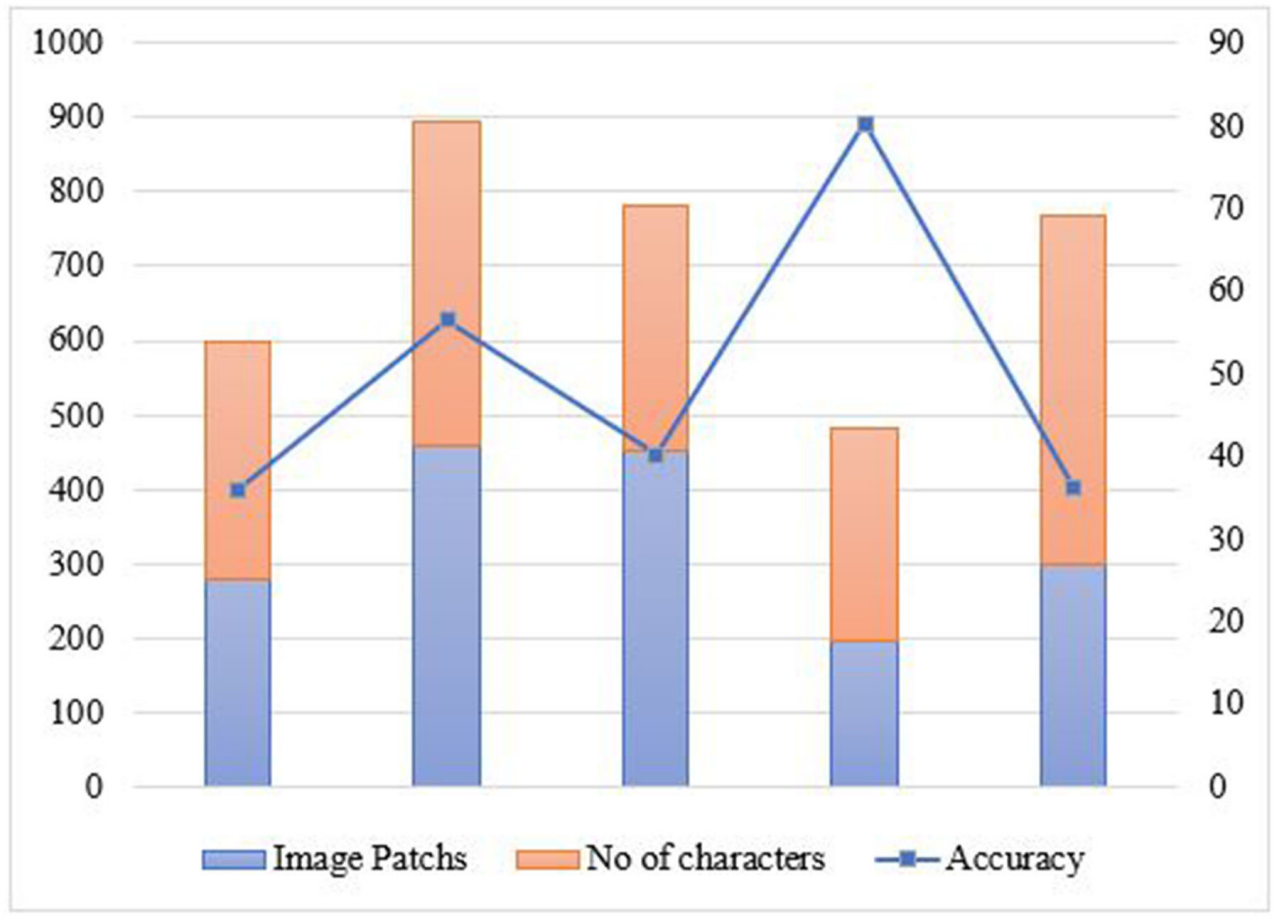
Performance accuracy for corresponding image data.

**Figure 8:**
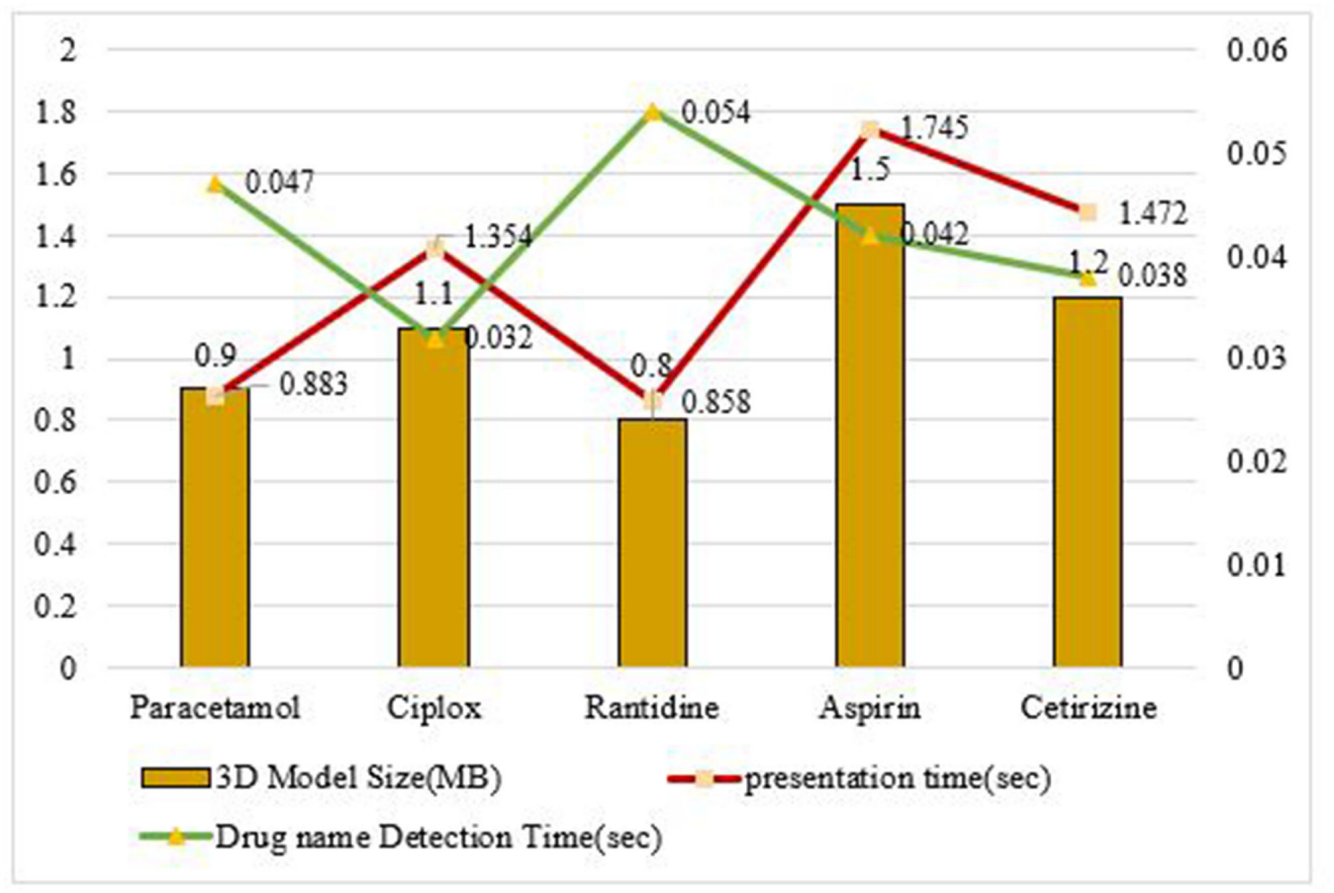
Response time for corresponding drug name detection and 3D model presentation.

Figure 7 shows the accuracy for recognition while testing different image patches over the proposed model. This system is tested on Android Studio by using a logger module. The testing had undergone different image patches and based on the characters recognized in the sources, the readings are noted accordingly. Figure 8 and Table 4 show the response time for recognition of drug name and presentation of the 3D model over the proposed system. The system is tested on Android Studio using the logger module and logcat.

**Table 4:** Response time-based analysis.

**3D model size**	**Sample drug used**	**Drug Name detection time (sec)**	**Presentation time for AR-based eye visual**
0.9 Mb	Paracetamol	0.047	0.883
1.1 Mb	Ciplox	0.032	1.354
0.8 Mb	Ranitidine	0.054	0.858
1.5 Mb	Aspirin	0.042	1.745
1.2 Mb	Cetirizine	0.038	1.472

## Conclusion and Future Scope

The proposed design was created by considering the issues looked at by individuals. The main concerns raised by the people during the fieldwork of the proposed system were that it is difficult to remember the medicinal names and that it would be an easy and comforting way to show the use of medicines visually. Those expectations are taken as the requirements for undertaking improvement. The proposed work helps out the people who cannot understand the use of the different kinds of medicines and helps in a visually and lively way by using AR, because of which the users will feel happy with the data they get past the application. The proposed system can process English texts and processing native languages is out of the scope of this system. A visual representation to show the use of medicines is considered best for every individual irrespective of whether they are educated or not. The expansion for this work is to create 3D models for the new medicines that are going to arrive in the market and extend the application to modify the classification of drugs based on user interaction. It can be extended to iOS-based mobile devices and to develop a voice-enabled control, for the navigation to access every feature of the application and it can be made available to all regional languages. The proposed work is limited to AR and it can be integrated with technologies like blockchain for providing more security and privacy.

## Data Availability Statement

The original contributions presented in the study are included in the article/supplementary material, further inquiries can be directed to the corresponding author.

## Author Contributions

CR: conceptualization and writing-original draft. GS: writing-review and editing. BG: conceptualization. ST: methodology. KM: investigation and validation. SK: formal analysis. JC-W: writing-review, editing, and formal analysis. All authors contributed to the article and approved the submitted version.

## Funding

This work was partially supported by the Western Norway University of Applied Sciences, Bergen, Norway.

## Conflict of Interest

The authors declare that the research was conducted in the absence of any commercial or financial relationships that could be construed as a potential conflict of interest.

## Publisher's Note

All claims expressed in this article are solely those of the authors and do not necessarily represent those of their affiliated organizations, or those of the publisher, the editors and the reviewers. Any product that may be evaluated in this article, or claim that may be made by its manufacturer, is not guaranteed or endorsed by the publisher.

## References

[1] Ashokkumar P Shankar SG Srivastava G Maddikunta PKR Gadekallu TR. A two-stage text feature selection algorithm for improving text classification. ACM Trans Asian Low Resour Lang Inf Process. (2021) 20:1–19. 10.1145/342578134706412

[2] Muñoz-Saavedra L Miró-Amarante L Domínguez-Morales M. Augmented and virtual reality evolution and future tendency. Appl Sci. (2020) 10:322. 10.3390/app10010322

[3] Yung R Khoo-Lattimore C. New realities: a systematic literature review on virtual reality and augmented reality in tourism research. Curr Issues Tourism. (2019) 22:2056–81. 10.1080/13683500.2017.1417359

[4] Bharath K Rupa Bhavani L chowdary M. Revelation of geospatial information using augmented reality. In: 2019 International Conference on Wireless Communications Signal Processing and Networking (WiSPNET). Chennai: IEEE (2021). p. 303–8.

[5] Reddy GT Reddy MPK Lakshmanna K Kaluri R Rajput DS Srivastava G. Analysis of dimensionality reduction techniques on big data. IEEE Access. (2020) 8:54776–88. 10.1109/ACCESS.2020.2980942

[6] Gadekallu TR Khare N Bhattacharya S Singh S Maddikunta PKR Srivastava G. Deep neural networks to predict diabetic retinopathy. J Ambient Intell Humaniz Comput. (2020) 1–14. 10.1007/s12652-020-01963-7

[7] Rizwan M Shabbir A Javed AR Srivastava G Gadekallu TR Shabir M. Risk monitoring strategy for confidentiality of healthcare information. Comput Electr Eng. (2022) 100:107833. 10.1016/j.compeleceng.2022.107833

[8] Reddy GT Reddy M Lakshmanna K Rajput DS Kaluri R Srivastava G. Hybrid genetic algorithm and a fuzzy logic classifier for heart disease diagnosis. Evol Intell. (2020) 13:185–96. 10.1007/s12065-019-00327-1

[9] Aggarwal R Singhal A. Augmented reality and its effect on our life. In: 2019 9th International Conference on Cloud Computing, Data Science & Engineering (Confluence). Noida: IEEE (2019). p. 510–5.

[10] Carmigniani J Furht B Anisetti M Ceravolo P Damiani E Ivkovic M. Augmented reality technologies, systems and applications. Multimed Tools Appl. (2011) 51:341–77. 10.1007/s11042-010-0660-6

[11] Maddikunta PKR Pham QV Prabadevi B Deepa N Dev K Gadekallu TR. Industry 5.0: a survey on enabling technologies and potential applications. J Ind Inf Integrat. (2021) 26: 100257. 10.1016/j.jii.2021.100257

[12] Wang S Qureshi MA Miralles-Pechuaán L Huynh-The T Gadekallu TR Liyanage M. Explainable AI for B5G/6G: technical aspects, use cases, and research challenges. arXiv preprint arXiv: 211204698. (2021).

[13] Wang W Qiu C Yin Z Srivastava G Gadekallu TR Alsolami F. Blockchain and PUF-based lightweight authentication protocol for wireless medical sensor networks. IEEE Internet Things J. (2021) 1–10. 10.1109/JIOT.2021.3117762

[14] Djenouri Y Belhadi A Srivastava G Lin JCW. Secure collaborative augmented reality framework for biomedical informatics. IEEE J Biomed Health Inf . (2021) 1–8. 10.1109/JBHI.2021.3139575 34971546

[15] Oufqir Z El Abderrahmani A Satori K. ARKit and ARCore in serve to augmented reality. In: 2020 International Conference on Intelligent Systems and Computer Vision (ISCV). Fez: IEEE (2020). p. 1–7.

[16] Huang J Kinateder M Dunn MJ Jarosz W Yang XD Cooper EA. An augmented reality sign-reading assistant for users with reduced vision. PLoS ONE. (2019) 14:e0210630. 10.1371/journal.pone.0210630 30650159 PMC6334915

[17] Guerrero E Lu MH Yueh HP Lindgren H. Designing and evaluating an intelligent augmented reality system for assisting older adults' medication management. Cogn Syst Res. (2019) 58:278–91. 10.1016/j.cogsys.2019.07.001

[18] Namysl M Konya I. Efficient, lexicon-free OCR using deep learning. In: 2019 International Conference on Document Analysis and Recognition (ICDAR). Sydney, NSW: IEEE (2019). p. 295–301.

[19] Qiao X Ren P Dustdar S Liu L Ma H Chen J. Web AR: A promising future for mobile augmented reality-State of the art, challenges, and insights. Proc IEEE. (2019) 107:651–66. 10.1109/JPROC.2019.2895105

[20] Lin H Yang P Zhang F. Review of scene text detection and recognition. Arch Comput Methods Eng. (2020) 27:433–54. 10.1007/s11831-019-09315-1

[21] Flores-Flores CD Sánchez-Cervantes JL Rodríguez-Mazahua L Colombo-Mendoza LO Rodríguez-González A. ARLOD: augmented reality mobile application integrating information obtained from the linked open drug data. In: Current Trends in Semantic Web Technologies: Theory and Practice. Mexico: Springer (2019). p. 269–92.

[22] Park YJ Ro H Lee NK Han TD. Deep-care: projection-based home care augmented reality system with deep learning for elderly. Appl Sci. (2019) 9:3897. 10.3390/app9183897

[23] Knopp S Klimant P Schaffrath R Voigt E Fritzsche R Allmacher C. Hololens ar-using vuforia-based marker tracking together with text recognition in an assembly scenario. In: 2019 IEEE International Symposium on Mixed and Augmented Reality Adjunct (ISMAR-Adjunct). Beijing: IEEE (2019). p. 63–4.

[24] Chaithanya C Manohar N Issac AB. Automatic text detection and classification in natural images. Int J Recent Technol Eng. (2019).

[25] Armengol-Estapé J Soares F Marimon M Krallinger M. PharmacoNER Tagger: a deep learning-based tool for automatically finding chemicals and drugs in Spanish medical texts. Genom Inform. (2019) 17:e15. 10.5808/GI.2019.17.2.e15 31307130 PMC6808625

[26] Rupa C Rao JR Babu PR. An efficient integrated ERP system using multilayer perceptron. In: Smart Intelligent Computing and Applications. Vijayawada: Springer (2019). p. 403–12.

[27] Molero D Schez-Sobrino S Vallejo D Glez-Morcillo C Albusac J. A novel approach to learning music and piano based on mixed reality and gamification. Multimed Tools Appl. (2021) 80:165–86. 10.1007/s11042-020-09678-9

[28] Harikrishna J Rupa C Gireesh R. Deep learning-based real-time object classification and recognition using supervised learning approach. In: Sentimental Analysis and Deep Learning. Nepal: Springer (2022). p. 129–39.

[29] Hasan MK Islam S Sulaiman R Khan S Hashim AHA Habib S. Lightweight encryption technique to enhance medical image security on internet of medical things applications. IEEE Access. (2021) 9:47731–42. 10.1109/ACCESS.2021.3061710

[30] Hasan MK Islam S Memon I Ismail AF Abdullah S Budati AK. A novel resource oriented DMA framework for internet of medical things devices in 5G network. IEEE Trans Ind Inform. (2022) 1–8. 10.1109/TII.2022.3148250

[31] Gadekallu TR Srivastava G Liyanage M Iyapparaja M Chowdhary CL Koppu S. Hand gesture recognition based on a harris hawks optimized convolution neural network. Comput Electr Eng. (2022) 100:107836. 10.1016/j.compeleceng.2022.107836

